# Oligonucleotide–Palladacycle Conjugates as Splice-Correcting Agents

**DOI:** 10.3390/molecules24061180

**Published:** 2019-03-26

**Authors:** Madhuri Hande, Osama Saher, Karin E. Lundin, C. I. Edvard Smith, Rula Zain, Tuomas Lönnberg

**Affiliations:** 1 Department of Chemistry, University of Turku, Vatselankatu 2, FIN-20014 Turku, Finland; nimamadhuri@gmail.com; 2 Department of Laboratory Medicine, Clinical Research Center, Karolinska Institutet, Karolinska University Hospital Huddinge, SE-141 86 Huddinge, Sweden; osama.ahmed@ki.se (O.S.) Karin.Lundin@ki.se (K.E.L.); Edvard.Smith@ki.se (C.I.E.S.); rula.zain@ki.se (R.Z.); 3 Department of Pharmaceutics and Industrial Pharmacy, Cairo University, Cairo 11562, Egypt; 4 Department of Clinical Genetics, Centre for Rare Diseases, Karolinska University Hospital, SE-171 76 Stockholm, Sweden

**Keywords:** oligonucleotide, organometallic, palladacycle, palladium, splice-correction

## Abstract

2’-*O*-Methylribo phosphorothioate oligonucleotides incorporating cyclopalladated benzylamine conjugate groups at their 5’-termini have been prepared and their ability to hybridize with a designated target sequence was assessed by conventional UV melting experiments. The oligonucleotides were further examined in splice-switching experiments in human cervical cancer (HeLa Luc/705), human liver (HuH7_705), and human osteosarcoma (U-2 OS_705) reporter cell lines. Melting temperatures of duplexes formed by the modified oligonucleotides were approximately 5 °C lower than melting temperatures of the respective unmodified duplexes. The cyclopalladated oligonucleotides functioned as splice-correcting agents in the HeLa Luc/705 cell line somewhat more efficiently than their unmodified counterparts. Furthermore, the introduction of this chemical modification did not induce toxicity in cells. These results demonstrate the feasibility of using covalently metalated oligonucleotides as therapeutic agents.

## 1. Introduction

After five decades of intense research, nucleic acids are finally maturing into useful drugs [1,2] in the form of antisense [3,4,5,6,7,8,9], antimiR [10,11,12,13,14,15], siRNA [16], and splice-switching oligonucleotides [17,18,19,20]. Modified oligonucleotides are needed in all of these approaches to overcome problems with cellular delivery and stability [21,22,23,24,25,26]. Any therapeutic oligonucleotide would also benefit from enhanced hybridization affinity for its target sequence. One way to achieve this is by metal coordination, as exemplified by numerous studies on metal-mediated base pairing [27,28,29,30]. A number of oligonucleotide conjugates of kinetically inert metal complexes, such as those of Pt(II) and Ru(II), have been described and in many cases shown to hybridize more efficiently than their unmodified counterparts [31,32,33,34,35,36,37]. We, on the other hand, have been interested in oligonucleotides covalently metalated with more labile transition metals and have recently reported on the synthesis and hybridization properties of the first oligonucleotides incorporating palladacyclic base moieties or conjugate groups [38,39].

While the potential of metal coordination to promote hybridization is well documented, we are not aware of any studies on the effect of covalent metalation on cellular uptake, toxicity, or biological activity of an oligonucleotide. The present article aims at partly filling this gap, describing splice-correction in vitro by phosphorothioate oligonucleotides featuring palladacyclic conjugate groups at their 5’-termini. The efficiency of splice-switching depends on the hybridization affinity of the therapeutic oligonucleotide for the aberrant splice site and could, hence, be improved by coordination of the covalently bound Pd(II) to nucleobases of the complementary strand. Splice-correction ability and toxicity were tested on three different human cell lines and the oligonucleotides were delivered by either lipofection or gymnosis. To the best of our knowledge this is the first use of covalently metalated oligonucleotides in a biological context and, hence, an important proof of concept.

## 2. Results

### 2.1. Oligonucleotide Synthesis

**Scheme 1:**
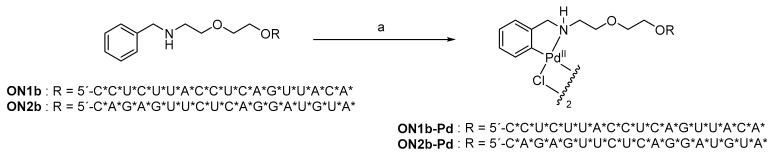
Cyclopalladation of oligonucleotides **ON1b** and **ON2b**. Reagents and conditions: (a) Li_2_PdCl_4_, MeCN, H_2_O, 25 °C, 24 h.

Oligonucleotide sequences used in the present study are listed in Table 1. The splice-switching oligonucleotides **ON1a**, **ON1b**, and **ON1b-Pd** and the respective negative controls **ON2a**, **ON2b**, and **ON2b-Pd** feature 2’-*O*-methylribo and phosphorothioate backbone modifications throughout their sequences. **ON3**, in turn, is an unmodified RNA oligonucleotide mimicking the aberrant splice site of the splice-correction assay (see below) [40]. The 5’-modified oligonucleotides **ON1b**, **ON1b-Pd**, **ON2b**, and **ON2b-Pd** were assembled on an automated DNA/RNA synthesizer using conventional phosphoramidite strategy. The 5’-terminal benzylamine moiety was introduced as the *N*-trifluorocetyl-protected building block described previously [39]. The sulfurization steps were carried out by treatment with 3-((dimethylaminomethylidene)amino)-3*H*-1,2,3-dithiazole-3-thione (DDTT) following the standard protocol. Conventional treatment with concentrated ammonia was employed for removal of base and phosphate protections and release of the oligonucleotides from solid support. Cyclopalladation of **ON1b** and **ON2b** was performed as described previously (Scheme 1) [39]. Finally, all modified oligonucleotides were purified by reverse-phase high performance liquid chromatography (RP-HPLC, Figures S1, S3, S5 and S7 in the Supporting Information), characterized by electrospray ionization mass spectrometry (ESI-MS, Figures S2, S4, S6 and S8 in the Supporting Information) and quantified UV spectrophotometrically.

### 2.2. Hybridization Studies

The 5’-terminal cyclopalladated benzylamine moiety has previously been shown to moderately enhance the hybridization of short DNA oligonucleotides [39]. To test whether this effect could be reproduced with considerably longer 2’-*O*-methyl-RNA oligonucleotides featuring a phosphorothioate backbone, melting profiles of duplexes formed by **ON1a**, **ON1b**, and **ON1b-Pd** with the native RNA target **ON3** were recorded. As a negative control, similar experiments were carried with **ON2a**, **ON2b**, and **ON2b-Pd**, lacking sequence complementarity to **ON3**. The pH of the samples was 7.4 (20 mM cacodylate buffer) and the ionic strength 0.10 M (adjusted with sodium perchlorate). Before measurement, the samples were annealed by heating to 90 °C and allowed to gradually cool down to room temperature.

**Figure 1:**
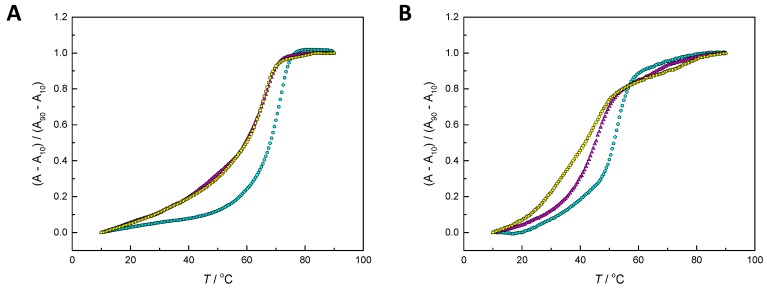
UV melting profiles for duplexes formed by the RNA **ON3** (**A**) and DNA **ON4** (**B**) targets with **ON1a** (cyan circles), **ON1b** (magenta triangles), and **ON1b-Pd** (yellow squares); pH = 7.4 (20 mM cacodylate buffer); [oligonucleotides] = 1.0 μM; *I*(NaClO_4_) = 0.10 M.

Duplexes **ON1a**•**ON3**, **ON1b**•**ON3**, and **ON1b-Pd**•**ON3** all exhibited sigmoidal melting curves with a single melting temperature at 71.1 ± 0.4, 66.7 ± 0.2, and 65.4 ± 0.6 °C, respectively (Figure 1A). In other words, the 5’-terminal benzylamine moiety was considerably destabilizing in both unmetalated and cyclopalladated form, in contrast with previous results on short DNA oligonucleotides. To test whether this unexpected result could be explained by different steric requirements of RNA–RNA and RNA–DNA double helices, the experiments were repeated with the DNA target sequence **ON4**. As expected, replacing the RNA target with a DNA target resulted in lower melting temperatures in all cases (52.4 ± 0.9, 45.8 ± 0.6, and 44.3 ± 0.8 for **ON1a**•**ON4**, **ON1b**•**ON4**, and **ON1b-Pd**•**ON4**, respectively) but the relative stabilities of the modified and unmodified duplexes remained largely unchanged. Nevertheless, the RNA–RNA duplexes **ON1a**•**ON3**, **ON1b**•**ON3**, and **ON1b-Pd**•**ON3** were all hybridized at the temperature of the splice-correction experiments (37 °C). As expected, none of the negative control oligonucleotides **ON2a**, **ON2b**, and **ON2b-Pd** formed a stable duplex with **ON3** (data not shown).

### 2.3. Reporter Cell Lines

Splice-switching ability of the cyclopalladated oligonucleotide **ON1b-Pd** and its unmetalated counterpart **ON1b** was tested on human cervical cancer (HeLa Luc/705), human osteosarcoma (U-2 OS_705) and human liver (HuH7_705) cell lines. Each of these cell lines carried the pLuc/705 splice-switching reporter—a luciferase-encoding gene interrupted by a mutated β-globin intron 2 [40,41,42]. The mutated intron presents an aberrant 5’ splice site which, in turn, activates a cryptic 3´ splice site, ultimately resulting in the translation of nonfunctional luciferase [40]. Hybridization of an oligonucleotide targeting the aberrant pre-mRNA results in splicing, which then yields the correct mRNA and restored luciferase activity, quantifiable by luminometry.

### 2.4. Splice-Correction Mediated by Lipofectamine 2000 Transfection of ONs

**Figure 2:**
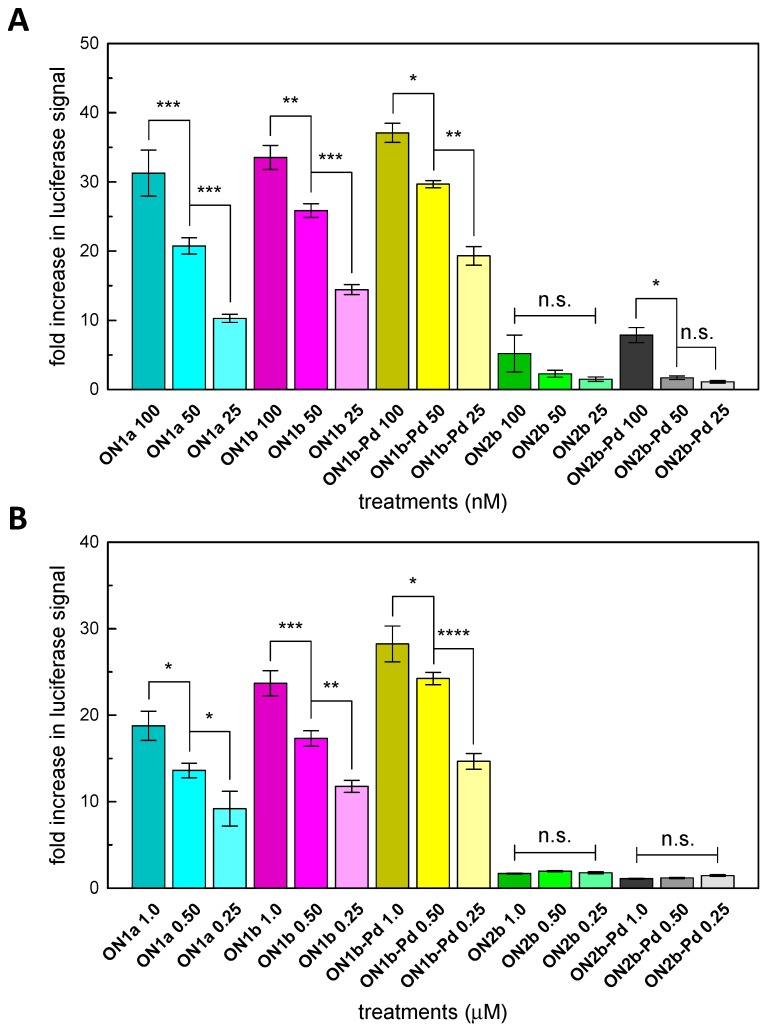
Restoration of Luciferase activity in HeLa Luc/705 cells by **ON1a** (cyan), **ON1b** (magenta), **ON1b-Pd** (yellow), **ON2b** (green), and **ON2b-Pd** (grey). The oligonucleotides were delivered by either lipofection (**A**) or gymnosis (**B**). Each column represents the mean with the standard error of the mean (SEM) of three (lipofection) or two (gymnosis) independent experiments (*n* ≥ 3). *P*-values were calculated by two-way ANOVA test and differences were statistically compared using post hoc Fisher’s LSD test (n.s. non-significant, * *p* ≤ 0.05, ** *p* ≤ 0.01, *** *p* ≤ 0.001 and **** *p* ≤ 0.0001).

Transfection by Lipofectamine 2000 has proven to be a robust delivery method for a wide range of cell types and modified oligonucleotides. In the present study, each of the three reporter cell lines was transfected with the modified oligonucleotides **ON1b** and **ON1b-Pd** and, for positive and negative controls, the unmodified oligonucleotide **ON1a** and the non-complementary oligonucleotides **ON2b** and **ON2b-Pd** (**ON2a** has been used previously and reported not to influence the splice-correction of the HeLa-705 construct [43]) following the previously reported protocol [44]. With the HeLa Luc/705 cell line, the experiments were carried out at three different oligonucleotide concentrations (25, 50 and 100 nM) and marked restoration of luciferase activity was observed by all the sequences complementary to the aberrant splice site. At each concentration, **ON1b** and, especially, **ON1b-Pd** were more efficient than **ON1**a with the HeLa Luc/705 cell line (Figure 2A). The non-complementary oligonucleotides **ON2b** and **ON2b-Pd** were inactive in all but the highest concentrations employed. As a control experiment, splice-correction in the HeLa Luc/705 cells was also investigated on the mRNA level at the highest oligonucleotide concentration (100 nM). The modified oligonucleotides **ON1b** and **ON1b-Pd** were again somewhat more efficient than their unmodified counterpart **ON1a** (Figure S9 in the Supporting Information). Curiously, considerable restoration of correct splicing was observed also with the non-complementary oligonucleotides **ON2b** and **ON2b-Pd**.

**Figure 3:**
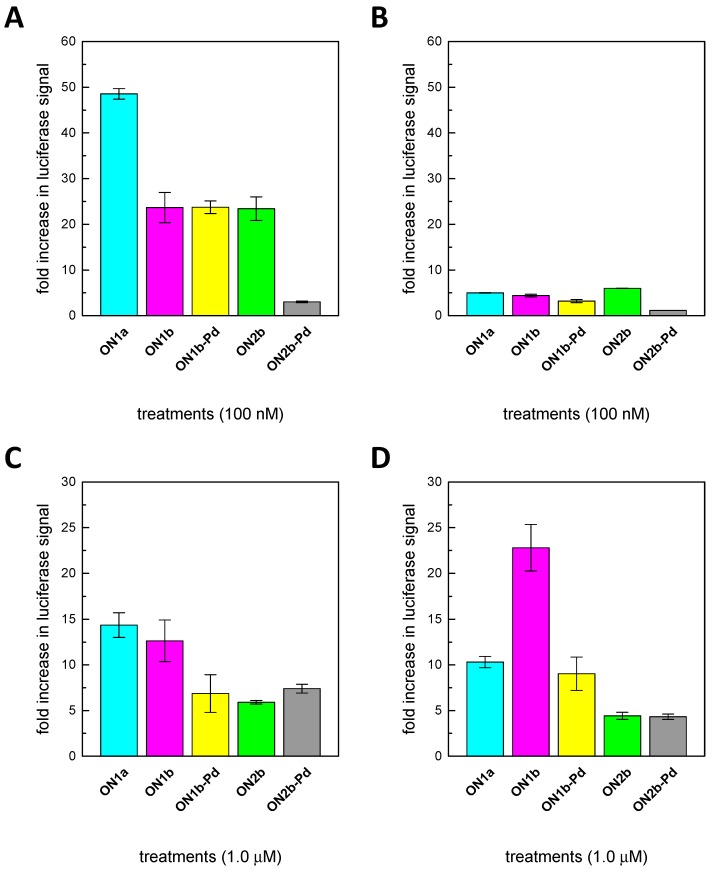
Restoration of Luciferase activity in HuH7_705 (**A**,**C**) and U-2 OS_705 (**B**,**D**) cells by **ON1a** (cyan), **ON1b** (magenta), **ON1b-Pd** (yellow), **ON2b** (green), and **ON2b-Pd** (grey). The oligonucleotides were delivered by either lipofection (**A**,**B**) or gymnosis (**C**,**D**). Each column represents the mean with the standard error of the mean (SEM) of at least three independent experiments (*n* ≥ 3).

With HuH7_705 and U-2 OS_705 cell lines, the experiments were carried out at a single concentration of 100 nM. With the HuH7_705 cell line, restoration of luciferase activity by the complementary sequences was comparable to that observed with the HeLa Luc/705 cell line at the highest oligonucleotide concentration (Figure 3A). In contrast, restoration of luciferase activity in the U-2 OS_705 cell line was very modest (Figure 3B). The most efficient splice-switching oligonucleotide was in both cases the unmodified **ON1a**. The palladated non-complementary control sequence **ON2b-Pd** was inactive whereas, unexpectedly, significant restoration of luciferase activity was observed on treatment with its unpalladated counterpart **ON2b**, in particular in the HuH7_705 cell line.

### 2.5. Splice-Correction Mediated by Gymnosis

Splice-correction by **ON1a**, **ON1b**, and **ON1b-Pd** and the negative controls **ON2b** and **ON2b-Pd** was also tested in the absence of any transfection agents (Figure 2B for the HeLa Luc/705 and Figure 3C,D for the HuH7_705 and U-2 OS_705 cell lines). As described above for transfection by Lipofectamine 2000, the experiments were carried out at three oligonucleotide concentrations (0.25, 0.50 and 1.0 μM) with the HeLa Luc/705 and at a single oligonucleotide concentration (1.0 μM) with the HuH7_705 and U-2 OS_705 cell lines. In most cases the splice-switching efficiency was lower in gymnosis than with transfection by Lipofectamine 2000, with the U-2 OS_705 cell line being an exception. In the HeLa Luc/705 cell line, a similar dose response pattern was observed as with lipofection, **ON1b-Pd** being again the most efficient splice-switching agent. In contrast, **ON1b-Pd** showed lower activity than **ON1b** or **ON1a** in the HuH7_705 and U-2 OS_705 cells. Curiously, the activity of the unpalladated oligonucleotide **ON1b** in the U-2 OS_705 cell line was considerably higher in gymnosis than when delivered by lipofection. These differences could reflect alterations in the abilities of the cyclopalladated and unmetalated oligonucleotides to penetrate into cells but the low levels of splice-correction achieved preclude firm conclusions.

### 2.6. Cell Viability

**Figure 4:**
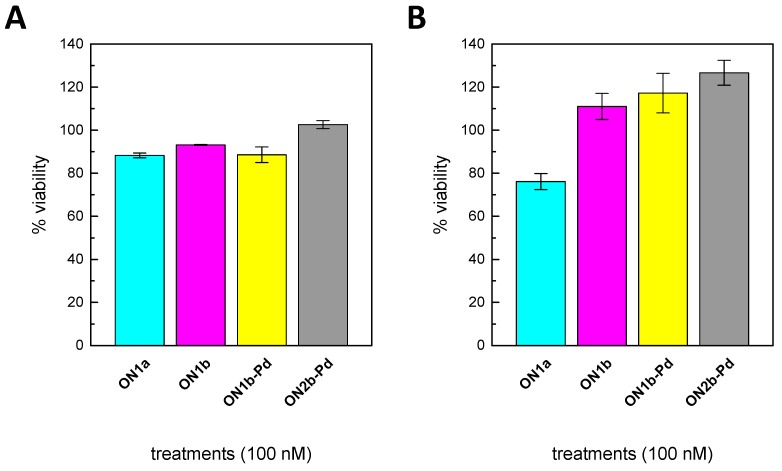
Viability of (**A**) HeLa Luc/705 and (**B**) HuH7_705 cells upon lipofection by oligonucleotides **ON1a** (cyan), **ON1b** (magenta), **ON1b-Pd** (yellow), and **ON2b-Pd** (grey). Each column represents the mean with the standard error of the mean (SEM) of two independent experiments (*n* = 2).

The viability of the HeLa Luc/705 and HuH7_705 cells after lipofection by the modified oligonucleotides was tested by a WST-1 cell proliferation assay. No marked toxicity was observed with any of the modified oligonucleotides, regardless of sequence or 5’-modification (Figure 4). With the HuH7_705 cell line, the number of viable cells after treatment with the modified oligonucleotides **ON1b**, **ON1b-Pd**, and **ON2b-Pd** was, in fact, somewhat higher than after treatment with the unmodified oligonucleotide **ON1a**.

## 3. Discussion

### 3.1. Hybridization Affinity of the Modified Oligonucleotides

The considerable thermal destabilization of duplexes **ON1b•ON3** and, especially, **ON1b-Pd•ON3** relative to **ON1a•ON3** was unexpected in light of our previous observation that the same terminal cyclopalladated benzylamine moiety stabilizes short DNA duplexes more than an additional Watson–Crick base pair [39]. Coordination of the covalently bound Pd(II) to the phosphorothioate backbone could explain the destabilization of **ON1b-Pd•ON3** but not the similar destabilization of the unpalladated **ON1b•ON3**. Another possible explanation is related to the different geometries of DNA–DNA and RNA–RNA double helices. The N donor of the benzylamine moiety is flanked by two sp^3^-hybridized carbon atoms that may be difficult to accommodate within the base stack and this problem would probably be more pronounced with the relatively rigid and tightly wound A-type double helix favored by RNA (while the benzylamine moiety itself is in a terminal position, the target sequence **ON3** features 2-nucleotide overhangs on both sides of the region complementary to the splice-switching oligonucleotides). RNA–DNA hybrid double helices, such as those formed by **ON1a**, **ON1b**, and **ON1b-Pd** with the DNA target **ON4**, also exhibit a mostly A-type conformation but are less rigid than RNA–RNA double helices. The similar destabilization of **ON1b•ON3**, **ON1b-Pd•ON3**, **ON1b•ON4**, and **ON1b-Pd•ON3** relative to the respective unmodified duplexes does not, hence, definitively rule out geometry of the double helix as the mechanism of this destabilization but nevertheless seems to argue against it.

### 3.2. Splice-Correction by the Modified Oligonucleotides

In the luciferase assay, the modified oligonucleotides **ON1b** and **ON1b-Pd** exhibited modestly higher splice-correction efficiency than their unmodified counterpart **ON1a** in the HeLa Luc/705 cell line while the opposite was true in the HuH7_705 cell lines, after lipofection at the highest oligonucleotide concentration employed (100 nM). A low but clear restoration of the luciferase signal was observed also after treatment with the non-complementary control **ON2b**. To exclude that this effect was just caused by a cellular activation leading to a general increase in reporter transcription, an RT-PCR was performed. Surprisingly, an increased restoration of the splicing could also be noticed at the mRNA level. A possible explanation of the effect of the modified control oligonucleotide would be that at higher concentrations the terminal benzylamine, which may act as an intercalator, would increase binding affinity of the partially complementary control oligonucleotide to the pre-mRNA. On the other hand, *T*_m_ measurements performed with the RNA target **ON3** appear to contradict this explanation. Still, it is only after delivery by lipofection at the highest concentration that this nonspecific increase in Luciferase was seen. In the gymnosis experiments, the correction pattern is in better agreement with the *T*_m_ measurements. It is also worth noting that palladation reduces the nonspecific effect of the control **ON2b** (Figure 3A,B). These apparent anomalies notwithstanding, our results show for the first time that covalently palladated oligonucleotides can be delivered to cells using standard methods, including gymnosis, and that within cells they are not toxic and they hybridize with the intended target sequence, paving way for future studies on improved organometallic oligonucleotides.

## 4. Materials and Methods

### 4.1. General Methods

Mass spectra were recorded on a Bruker Daltonics micrOTOF-Q mass spectrometer (Bruker, Billerica, MA, USA). Freshly distilled triethylamine was used for preparation of the HPLC elution buffers. The other chemicals, including oligonucleotides **ON1a**, **ON2a**, **ON3**, and **ON4**, were commercial products and used as received.

### 4.2. Oligonucleotide Synthesis

Modified phosphorothioate oligonucleotides **ON1b**, **ON1b-Pd**, **ON2b**, and **ON2b-Pd** were synthesized on an Applied Biosystems 3400 (Applied Biosystems, Waltham, MA, USA) automated DNA/RNA synthesizer by conventional phosphoramidite strategy, with 5-(benzylthio)-1*H*-tetrazole as the activator and 3-((dimethylaminomethylidene)amino)-3*H*-1,2,3-dithiazole-3-thione (DDTT) as the sulfurizing agent. A coupling time of 300 s was employed for both the commercial 2´-*O*-methylribonucleoside building blocks as well as the *N*-trifluorocetyl-protected benzylamine building block [39]. After chain assembly, removal of base and phosphate protections and release of the oligonucleotides from solid support was accomplished by treatment with 25% aqueous ammonia at 55 °C for 16 h. Cyclopalladation of **ON1b** and **ON2b** was carried out by incubating 115 nmol of the oligonucleotide and 173 nmol of Li_2_PdCl_4_ in a mixture of H_2_O (454 μL) and MeCN (454 μL) at 25 °C for 24 h. Finally, all modified oligonucleotides were purified by reversed-phase high performance liquid chromatography (RP- HPLC) on a Hypersil ODS C18 column (250 × 4.6 mm, 5 μm, Thermo Fisher Scientific, Waltham, MA, USA) eluting with a linear gradient (0 to 50% over 30 min) of MeCN in 50 mM aqueous triethylammonium acetate. The purified oligonucleotides were characterized by electrospray ionization mass spectrometry (ESI-MS). Products **ON1b-Pd** and **ON2b-Pd** were contaminated by approximately 15% of the unpalladated starting materials **ON1b** and **ON2b** but the purity was deemed acceptable for the hybridization and splice-switching studies. The oligonucleotides were quantified by UV spectrophotometry using molar absorptivities calculated by an implementation of the nearest-neighbors method. Contribution of the benzylamine moiety, whether free or cyclopalladated, was assumed to be negligible.

### 4.3. UV Melting Temperature Measurements

UV melting profiles were recorded on a PerkinElmer Lambda 35 UV-Vis spectrometer equipped with a Peltier temperature control unit (PerkinElmer, Waltham, MA, USA). The samples contained the appropriate oligonucleotides in 1.0 μM concentration at pH = 7.4 (20 mM cacodylate buffer) and *I* = 0.10 M (adjusted with NaClO_4_). Before measurement, the samples were annealed by heating to 90 °C and then allowing to cool slowly to room temperature. The denaturation and renaturation curves were obtained by monitoring the absorbance at *λ* = 260 nm as a function of temperature (10–90 °C, ramping by 0.5 °C min^−1^), sampling at 0.5 °C intervals. The melting temperatures were determined as inflection points on the denaturation and renaturation curves.

### 4.4. Cell Lines and Culture Conditions

The three reporter cell lines, HeLa Luc/705 (human cervical cancer cells), HuH7_705 (human liver cells), and U-2 OS_705 (human osteosarcoma cells) [41], were cultured and maintained in high glucose Dulbecco’s modified Eagle’s medium (DMEM) supplemented with 10% fetal bovine serum (FBS) at 37 °C in a humidified incubator with 5% CO_2_.

### 4.5. Transfection with Lipofectamine 2000

Cells were seeded at a density of 1.1 × 10^4^ per well in white TC-Treated 96-well plates (Corning^®^, Gothenburg, Sweden) one day before transfection, to reach cell confluency of approximately 70–80%. Lipofectamine 2000/oligonucleotide (25, 50 or 100 nM) complexes were prepared in OptiMEM^®^ for transfection according to the manufacturer’s protocol. Before adding the complexes to cells, culture media were removed and 100 μL of complexes were added per well. After 4 h, the complexes were replaced with complete media with 10% FBS (100 μL per well). Cells were incubated at 37 °C in a humidified incubator with 5% CO_2_ for another 20 h before analysis.

### 4.6. Gymnosis Experiments

Cells were seeded at a density of 5 × 10^3^ per well in white TC-treated 96-well plates. One day after seeding, media were replaced with fresh media supplemented with 9 mM of CaCl_2_, together with or without appropriate amount of oligonucleotide to achieve 1 μM final concentration per well, using the Hori et al. protocol [45]. The medium was later replaced with fresh medium supplemented with 9 mM of CaCl_2_ after 48–72 h. Cells were incubated at 37 °C in a humidified incubator with 5% CO_2_ and harvested for luciferase measurements 7 days after initial treatment.

### 4.7. Luciferase Assay

A modification of the previously reported protocol for the luciferase assay was followed [41]. The medium was removed, and the cells lysed in 25 μL of 0.1% Triton-X 100 in 1X PBS per well in a 96-well plate. After lysis of the cells, 5 μL were used to determine total protein quantity by DC Protein Assay (BioRad). The remaining 20 μL of lysates were mixed with 50 μL of the luciferase reagent (Promega) added via injector. The relative light units (RLU) of luciferase were measured (GloMax^®^ 96 Microplate Luminometer machine, Promega, Sweden) with 10 s integration time and 2 s delay between injection and measurement. The values were divided by the total protein quantities determined and normalized to untreated well values. Final results are represented as fold increase in luciferase activity.

### 4.8. RNA Expression Analysis

For determination of expression levels of corrected luciferase mRNA, total RNA was isolated from the cells using the RNeasy plus kit. Three nanograms of isolated RNA were used in each RT-PCR reaction using ONE STEP RT-PCR kit. The total volume was 20 μL and the sequences of the forward and reverse primers were 5′-TTGATATGTGGATTTCGAGTCGTC-3′ and 5′-TGTCAATCAGAGTGCTTTTGGCG-3′, respectively. The program for the RT-PCR was as follows: 35 min at 55 °C and 15 min at 95 °C for the reverse transcription, followed directly by 30 cycles of PCR (30 s at 94 °C, 30 s at 55 °C, and 30 s at 72 °C) and, finally, 10 min at 72 °C for the final extension. The PCR products were analyzed in a 1.5% agarose gel in 1 × TBE buffer and visualized by SYBR Gold staining. Documentation of gels was done with the Versadoc imaging system equipped with a cooled CCD camera (BioRad, Hercules, CA, USA). Band intensities were analyzed with the Quantity One software and the percentage of corrected mRNA was calculated by normalization against the sum of band intensities of corrected and uncorrected bands.

### 4.9. Cell Viability Assay

The viability of cells after lipofection with the modified oligonucleotides was assessed with the WST-1 assay (Roche, Germany). Cells were seeded and transfected as described above. 24 h after transfection, the media were replaced with fresh media supplemented with WST-1 reagent (dilution 1:10 with H_2_O). After addition of media with WST-1 reagent, the cells were further incubated for 2 h at 37 °C in a humidified incubator with 5% CO_2_ according to the manufacturer’s protocol. Absorbance measurements were carried out on a SpectraMAX i3x Western Blot Imager (Molecular Devices, San José, CA, USA) at *λ* = 450 nm with a reference wavelength of 650 nm. Values were expressed as the ratio of the absorbance at 450 nm of the treated cells to the untreated cells.

### 4.10. Data analysis

Data are expressed as mean with standard error of the mean (SEM). Statistical significance was determined by one- or two-way analysis of variance (ANOVA) followed by individual comparisons using Fisher’s least significant difference test. In all cases, *p* < 0.05 was considered significant.

## 5. Conclusions

Phosphorothioate oligonucleotides bearing a 5’-terminal cyclopalladated benzylamine moiety can be delivered to cells in vitro using standard protocols. The modified oligonucleotides were non-toxic and effected splice-correction by hybridization with the designated aberrant splice site. In the HeLa Luc/705 cell line, the modified oligonucleotides were more efficient as splice-correcting agents than their unmodified counterparts. The results thus demonstrate for the first time the feasibility of using covalently metalated oligonucleotides as therapeutic agents.

## Figures and Tables

**Table 1:** Oligonucleotides used in this study.

Oligonucleotide	Sequence ^1^
**ON1a**	5′-C*C*U* C*U*U* A*C*C* U*C*A* G*U*U* A*C*A*-3′
**ON1b**	5′-B- C*C*U* C*U*U* A*C*C* U*C*A* G*U*U* A*C*A*-3′
**ON1b-Pd**	5′-B^Pd^- C*C*U* C*U*U* A*C*C* U*C*A* G*U*U* A*C*A*-3′
**ON2a**	5´-C*A*G* A*G*U* U*C*U* C*A*G* G*A*U* G*U*A*-3′
**ON2b**	5′-B- C*A*G* A*G*U* U*C*U* C*A*G* G*A*U* G*U*A*-3′
**ON2b-Pd**	5′-B^Pd^- C*A*G* A*G*U* U*C*U* C*A*G* G*A*U* G*U*A*-3′
**ON3**	5′-AUU GUA ACU GAG GUA AGA GGU U-3′
**ON4**	5′-att gta act gag gta aga ggt t-3′

^1^ B refers to unmetalated and B^Pd^ to cyclopalladated benzylamine residue. Starred letters refer to 2’-*O*-methylribo phosphorothioates, all other uppercase letters to natural ribonucleotides and lowercase letters to deoxyribonucleotides.

## Supplementary Materials

The following are available online at https://www.mdpi.com/1420-3049/24/6/1180/s1, Figure S1: HPLC trace of oligonucleotide **ON1b**, Figure S2: Mass spectrum of oligonucleotide **ON1b**, Figure S3: HPLC trace of oligonucleotide **ON2b**, Figure S4: Mass spectrum of oligonucleotide **ON2b**, Figure S5: HPLC trace of oligonucleotide **ON1b-Pd**, Figure S6: Mass spectrum of oligonucleotide **ON1b-Pd**, Figure S7: HPLC trace of oligonucleotide **ON2b-Pd**, Figure S8: Mass spectrum of oligonucleotide **ON2b-Pd**, Figure S9: A gel of RT-PCR of luciferase mRNA and efficiency of splice-correction in HeLa Luc/705 cells.
