# Supplementary material for: Oligonucleotide–Palladacycle Conjugates as Splice-Correcting Agents
Source: Molecules. 2019 Mar 26;24(6):1180. doi: 10.3390/molecules24061180 (PMC6470670; doi:10.3390/molecules24061180)
Supplement: Supplementary file 1 [file molecules-24-01180-s001.pdf]

Supporting Information for

# Oligonucleotide-Palladacycle Conjugates as Splice Correcting Agents

Madhuri Hande [1], Osama Saher [2,3], Karin E. Lundin [2], C. I. Edvard Smith [2], Rula Zain [2,4] and Tuomas Lönnberg [1,*]

[1] Department of Chemistry, University of Turku, Vatselankatu 2, FIN-20014 Turku, Finland
[2] Department of Laboratory Medicine, Clinical Research Center, Karolinska Institutet, Karolinska University Hospital Huddinge, SE-141 86 Huddinge, Sweden
[3] Department of Pharmaceutics and Industrial Pharmacy, Cairo University, Cairo, Egypt
[4] Department of Clinical Genetics, Centre for Rare Diseases, Karolinska University Hospital, SE-171 76 Stockholm, Sweden
* Correspondence: tuanlo@utu.fi; Tel.: +358-29-450-3191

## Contents

Figure S1. HPLC trace of oligonucleotide ON1b ..... S2

Figure S2. Mass spectrum of oligonucleotide ON1b ..... S2

Figure S3. HPLC trace of oligonucleotide ON2b ..... S3

Figure S4. Mass spectrum of oligonucleotide ON2b ..... S3

Figure S5. HPLC trace of oligonucleotide ON1b-Pd ..... S4

Figure S6. Mass spectrum of oligonucleotide ON1b-Pd ..... S4

Figure S7. HPLC trace of oligonucleotide ON2b-Pd ..... S5

Figure S8. Mass spectrum of oligonucleotide ON2b-Pd ..... S5

Figure S9. A gel of RT-PCR of luciferase mRNA and efficiency of splice correction in HeLa Luc/705 cells ..... S6

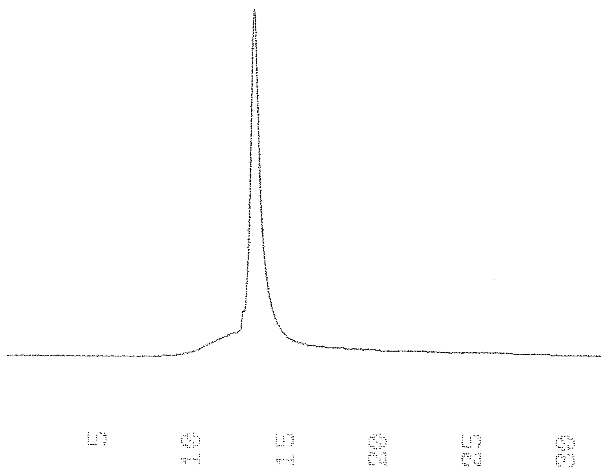

Figure S1. HPLC trace of oligonucleotide ON1b; Hypersil ODS C18 column (250 × 4.6 mm, 5 μm); flow rate = 1.0 mL min$^{-1}$; linear gradient (0 to 50% over 30 min) of MeCN in 50 mM aqueous triethylammonium acetate.

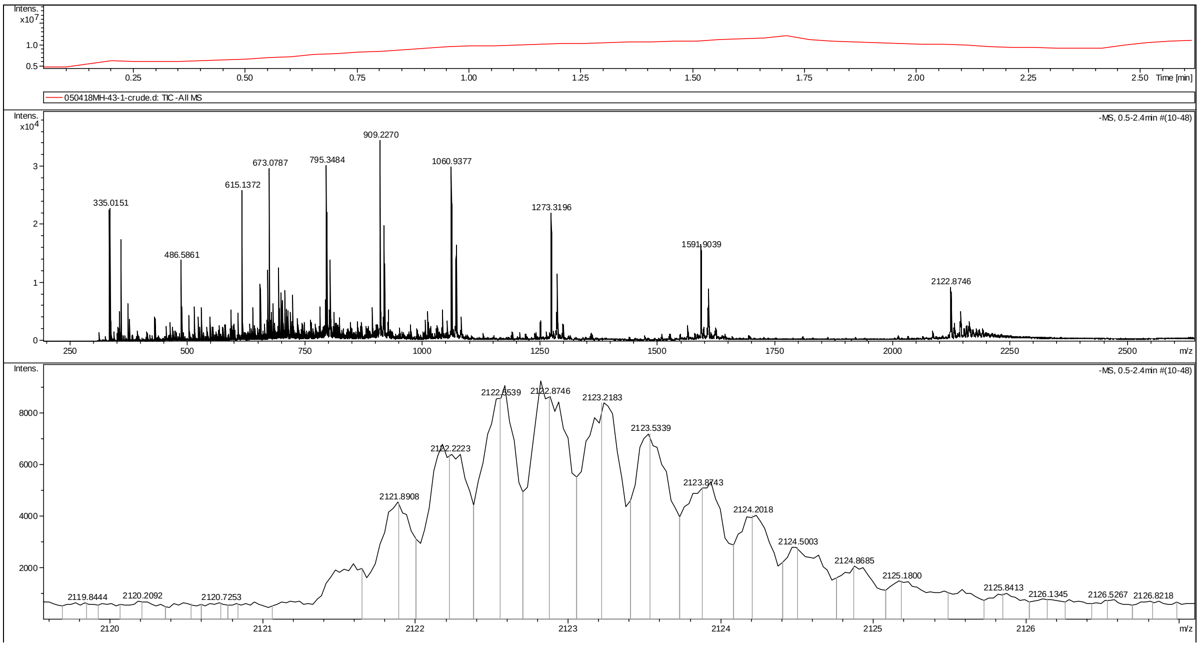


Figure S2. Mass spectrum of oligonucleotide ON1b; m/z calcd for $C_{196}H_{260}N_{59}O_{112}P_{18}S_{18}$: 2122.2265; found: 2122.2223 $[M - 3H]^{3-}$.

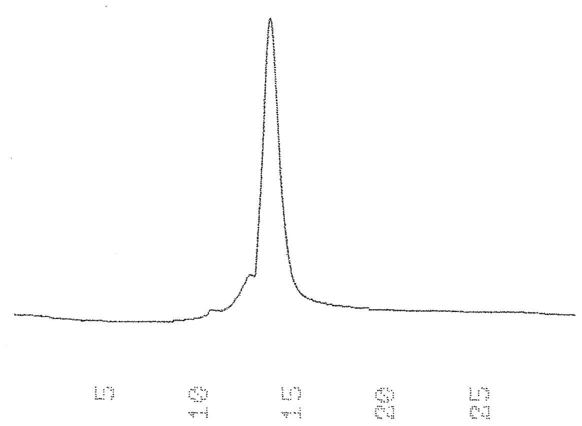

Figure S3. HPLC trace of oligonucleotide ON2b; Hypersil ODS C18 column (250 × 4.6 mm, 5 μm); flow rate = 1.0 mL min$^{-1}$; linear gradient (0 to 50% over 30 min) of MeCN in 50 mM aqueous triethylammonium acetate.

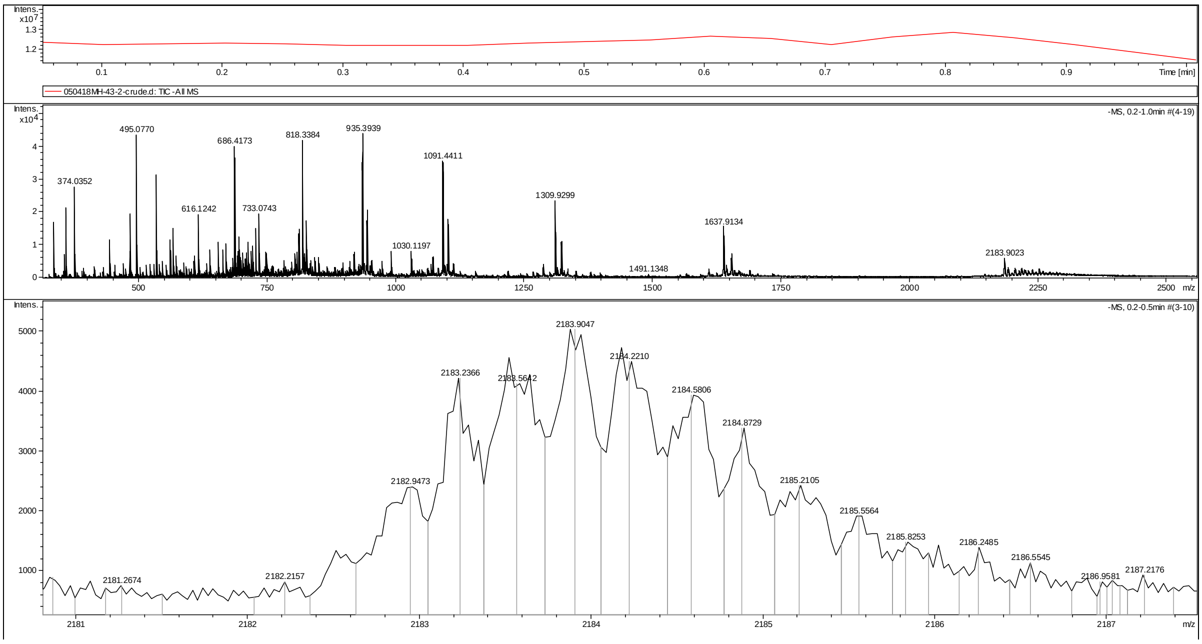


Figure S4. Mass spectrum of oligonucleotide ON2b; m/z calcd for $C_{201}H_{261}N_{70}O_{110}P_{18}S_{18}$: 2183.2438; found: 2183.2366 $[M - 3H]^{3-}$.

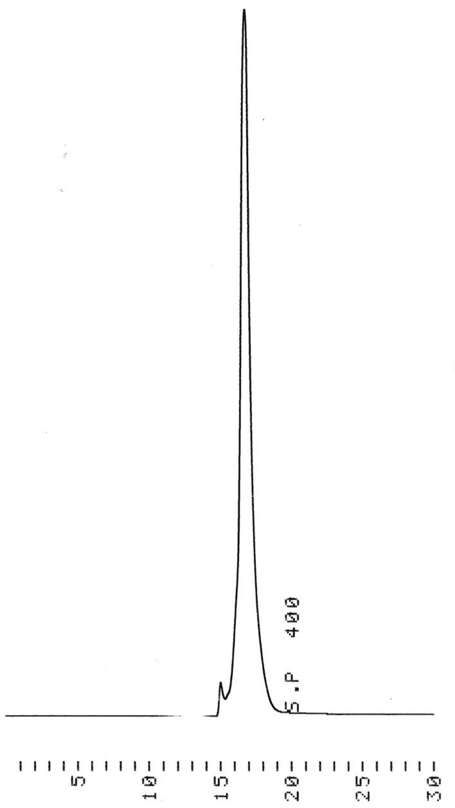

Figure S5. HPLC trace of oligonucleotide ON1b-Pd; Hypersil ODS C18 column (250 × 4.6 mm, 5 μm); flow rate = 1.0 mL $min^{-1}$; linear gradient (0 to 50% over 30 min) of MeCN in 50 mM aqueous triethylammonium acetate.

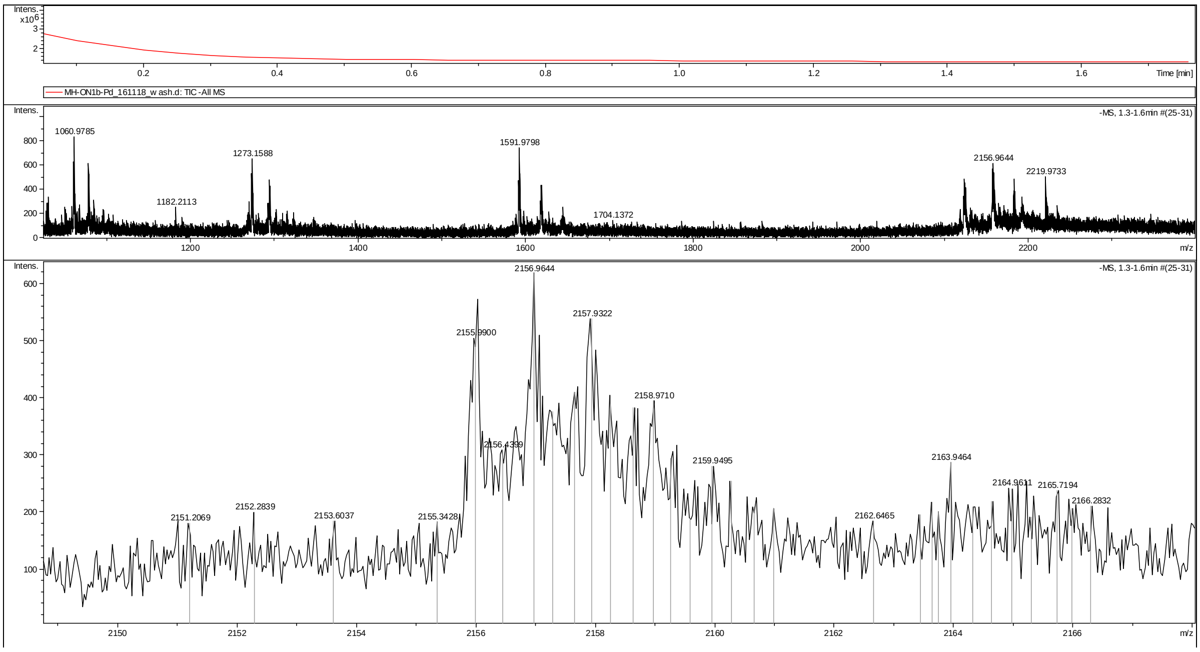


Figure S6. Mass spectrum of oligonucleotide ON1b-Pd; m/z calcd for $C_{196}H_{258}N_{59}O_{112}P_{18}S_{18}Pd$: 2156.8558; found: 2156.9644 $[M – 4H]^{3-}$.

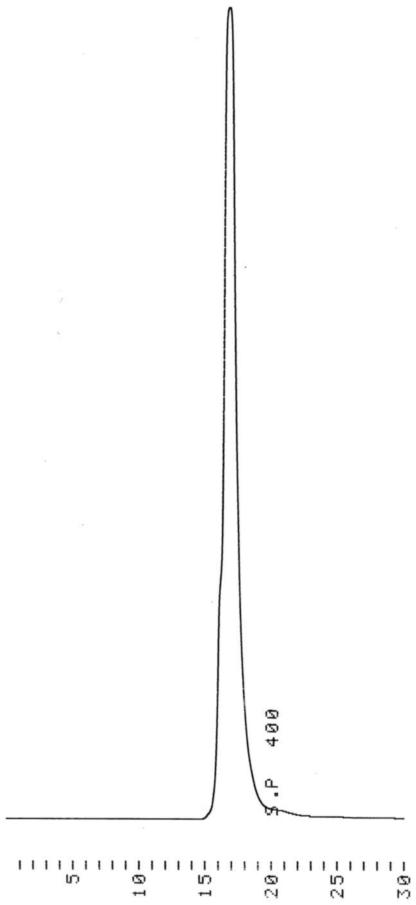


Figure S7. HPLC trace of oligonucleotide ON2b-Pd; Hypersil ODS C18 column (250 × 4.6 mm, 5 μm); flow rate = 1.0 mL $min^{-1}$; linear gradient (0 to 50% over 30 min) of MeCN in 50 mM aqueous triethylammonium acetate.

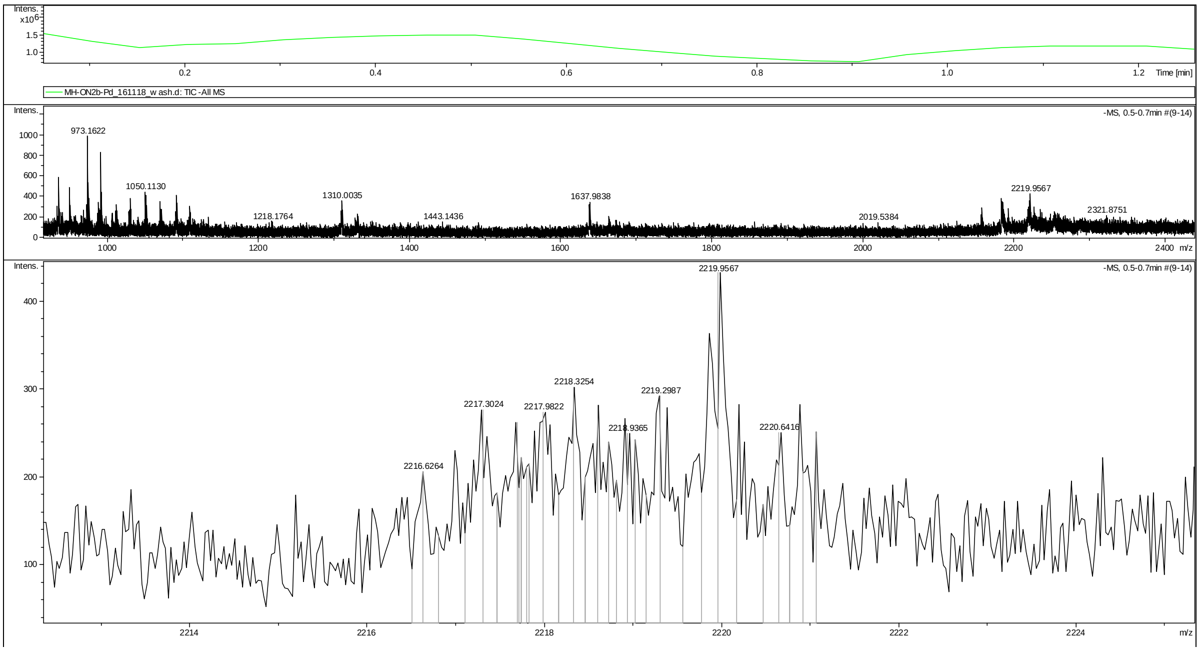


Figure S8. Mass spectrum of oligonucleotide ON2b-Pd; m/z calcd for $C_{201}H_{259}N_{70}O_{110}P_{18}S_{18}Pd$: 2217.8731; found: 2217.9622 $[M - 4H]^{3-}$.

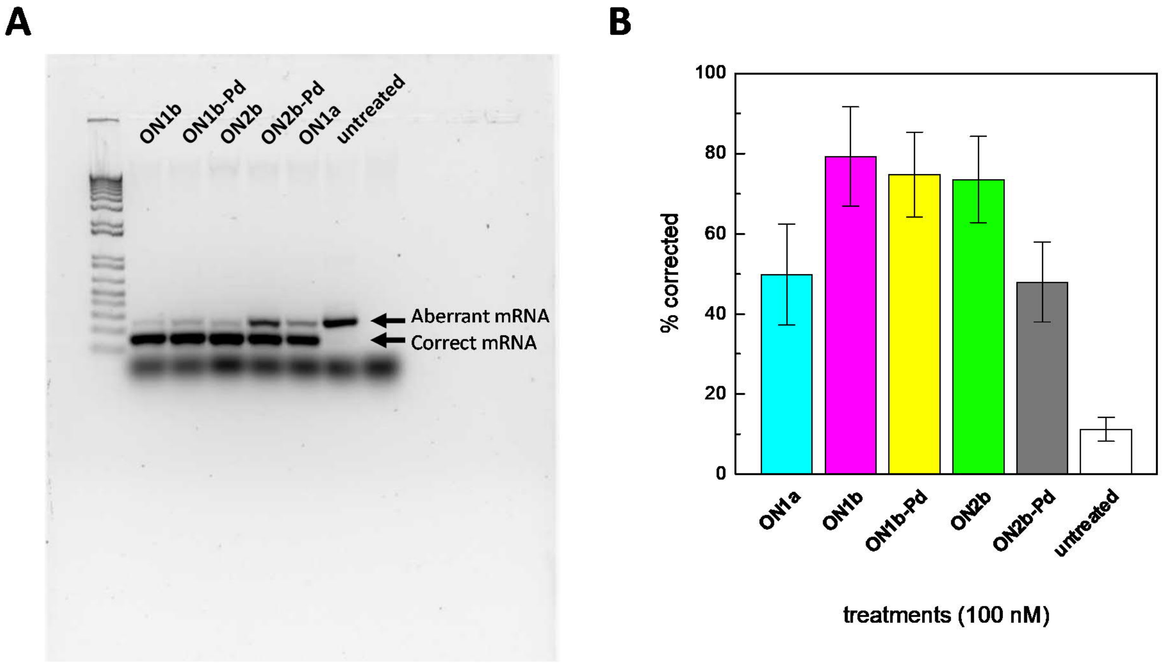


Figure S9. A gel of RT-PCR of luciferase mRNA (A) and efficiency of splice correction (B) in HeLa Luc/705 cells by oligonucleotides ON1a (cyan), ON1b (magenta), ON1b-Pd (yellow), ON2b (green) and ON2b-Pd (grey), as well as without any treatment (white) . The oligonucleotides were delivered by lipofection. Each column represents the mean with the standard error of the mean (SEM) of at least three independent experiments ($n \geq 3$).
